# Functional Hydrogels and Their Applications in Craniomaxillofacial Bone Regeneration

**DOI:** 10.3390/pharmaceutics15010150

**Published:** 2022-12-31

**Authors:** Yi Yu, Tingting Yu, Xing Wang, Dawei Liu

**Affiliations:** 1 Department of Orthodontics, Peking University School and Hospital of Stomatology, Beijing 100081, China; 2 National Clinical Research Center for Oral Diseases & National Engineering Laboratory for Digital and Material Technology of Stomatology, Beijing 100081, China; 3 Beijing Key Laboratory of Digital Stomatology, Beijing 100081, China; 4 Beijing National Laboratory for Molecular Sciences, Institute of Chemistry, Chinese Academy of Sciences, Beijing 100190, China

**Keywords:** hydrogel, craniomaxillofacial bone, bone regeneration, stimuli-responsive system, tissue engineering, delivery system

## Abstract

Craniomaxillofacial bone defects are characterized by an irregular shape, bacterial and inflammatory environment, aesthetic requirements, and the need for the functional recovery of oral–maxillofacial areas. Conventional clinical treatments are currently unable to achieve high-quality craniomaxillofacial bone regeneration. Hydrogels are a class of multifunctional platforms made of polymers cross-linked with high water content, good biocompatibility, and adjustable physicochemical properties for the intelligent delivery of goods. These characteristics make hydrogel systems a bright prospect for clinical applications in craniomaxillofacial bone. In this review, we briefly demonstrate the properties of hydrogel systems that can come into effect in the field of bone regeneration. In addition, we summarize the hydrogel systems that have been developed for craniomaxillofacial bone regeneration in recent years. Finally, we also discuss the prospects in the field of craniomaxillofacial bone tissue engineering; these discussions can serve as an inspiration for future hydrogel design.

## 1. Introduction

Craniomaxillofacial bone tissue consists of the cranial bone, the maxillo-mandibular bone, and the alveolar bone, which is quite specific among the bone tissues of the whole body. Inflammation, tumors, trauma, and maxillofacial deformities can lead to defects in craniofacial bone tissue [1,2]. Craniomaxillofacial bone defects are more difficult to treat due to the irregular shape of the defect sites and the poor healing environment with bacteria and inflammation, as well as the higher aesthetic requirements of the patient and the need for the functional recovery of the oral–maxillofacial areas. Currently, autologous bone grafting is widely used for clinical repair, but autologous bone repair has the disadvantages of being a limited source, causing secondary injury to the patient, and not being able to aesthetically repair irregular defects [3]. In this case, the field of craniomaxillofacial bone defect repair remains a major challenge.

Hydrogels are suitable for applications in the maxillofacial region, because they exhibit degradability, modifiability, and good biocompatibility, facilitating the healing of bone defects and restoring maxillofacial aesthetics [4]. Hydrogels are a class of synthetic or natural polymeric cross-linked, highly water-containing 3D mesh materials that have an important status in the biomedical field. First, the strong in situ forming ability of hydrogels can repair craniomaxillofacial irregularly shaped bone defects very well. Secondly, the mechanical properties of the hydrogels can be adjusted by optimizing the degree of polymerization, cross-linking methods, preparation methods, etc., such as the regulation of the rigidity [5] and pore size [6] of hydrogel systems. In addition, hydrogels are highly water rich, often with a water content of more than 70%. Water, which makes up the largest part of its components, gives hydrogels an interfacial composition similar to that of tissues, providing them with a high degree of biocompatibility and biosafety [7]. These properties of hydrogels, including cross-linked networks and high-water content, provide excellent performance for loading cells and various types of drugs. Moreover, as scaffold materials, hydrogels with good cell loading capacity are becoming an indispensable component of tissue engineering, which can provide an environment that resembles the natural bone structure for bone tissue healing and can better promote bone repairing [8,9].

This paper summarizes and discusses the unique properties of hydrogels, which enable the intelligent delivery of cargo in the field of craniofacial bone tissue repair and advancements in the development of hydrogels for craniomaxillofacial bone regeneration applications. Previous reviews have summarized the preparation of functional hydrogels [10], hydrogel forms for bone tissue engineering (e.g., micro/nano hydrogels and injectable hydrogels) [11], hydrogel-delivered drugs for bone tissue engineering applications [12], and hydrogels that can be used to repair bone defects by bioprinting [13,14]. We focus on translational medicine, summarizing the structure and properties of hydrogels suitable for application in craniomaxillofacial bone tissue engineering, and current advances. More importantly, we provide critical perspectives on the issues that need to be considered when designing hydrogels for application in craniomaxillofacial bone defects, the challenges that may be faced in hydrogel development, and proposed approaches to address these issues based on an understanding of hydrogels.

## 2. Hydrogels: Distinctive Properties for Craniomaxillofacial Bone Regeneration

**Figure 1:**
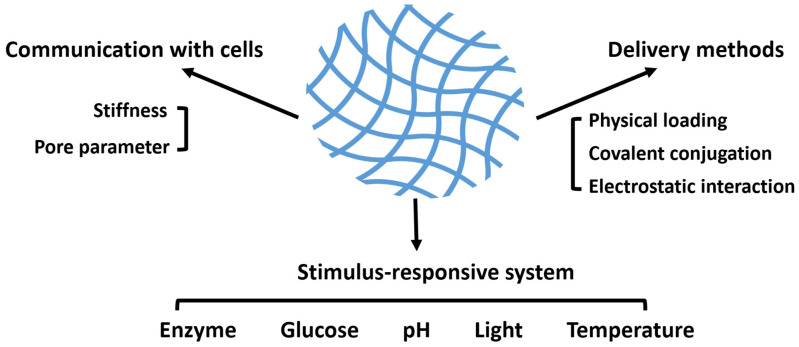
General scheme of the essential parameters of hydrogels applied in craniomaxillofacial bone regeneration. Physical and chemical properties, such as stiffness and mesh size, endow hydrogels with the ability to communicate with cells and to transport a variety of cargoes. Furthermore, in order to obtain a better effect of bone repairment, several potential stimulus-responsive systems are listed.

The in situ forming ability and plasticity of hydrogels enable the aesthetic repair of craniomaxillofacial bone defects. The high hydration and interlinked network of hydrogels give them good biocompatibility and physical properties, making hydrogels an excellent vehicle for transporting cells [15,16,17,18]. In addition, one of the major advantages of hydrogels is that their composition can be modified to obtain drug-carrying properties for different types of drugs. The hydrogel loading of drugs mainly includes physical encapsulation, covalent anchoring and electrostatic adsorption [19]. Different drugs are loaded into the hydrogel system in different specific ways, giving the hydrogel the ability to respond intelligently to the physiological processes of healing of many types of complex bone tissues in the craniomaxillofacial region, providing a “smarter” means of craniomaxillofacial bone tissue repair (Figure 1).

### 2.1. Unique Physicochemical Properties Enabling Communication with Cells

**Figure 2:**
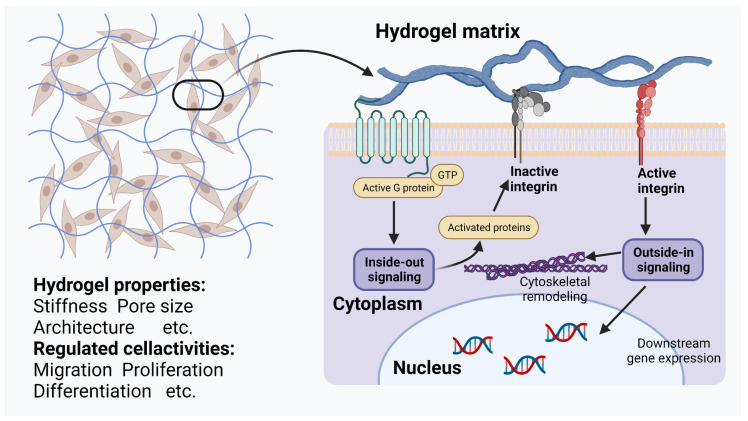
Schematic representation of the communication between hydrogel and the cell. Modulation of various modifiable physicochemical properties of the hydrogel can act on cell surface receptors (such as α1β1 and α2β1 integrins [23] and CD44 [24], and depends on the type and physicochemical properties of the hydrogel) and thereby modulate intracellular signal transduction pathways (such as STAT3 [24]). This figure was created with BioRender, accessed on 1 December 2022 (https://biorender.com/).

Hydrogels, which are implanted as local scaffolds to promote bone tissue healing in situ, have a variety of modifiable physicochemical properties that, by virtue of their unique and individualized properties, allow them to communicate with the cells in which they are loaded or with cells in the surrounding environment, activating intracellular signaling pathways and promoting bone regeneration (Figure 2). Therefore, when implanted at the site of an injury, hydrogels should be carefully tailored.

First, the pore size in the hydrogel needs to be controlled. Hung et al., reported the presence of cell-surface integrin-mediated signaling between cells and the surrounding environment during cell migration [20]. Stem cells in small pore volume (30 μm^2^) gelatin-based scaffolds have previously been shown to exhibit a higher degree of osteogenic differentiation compared with larger scaffolds (100 μm^2^) [21], which is in line with the understanding of skeletal histology—dense calcium phosphate crystals self-assemble around a very small number of mature osteocytes. Additionally, the appropriate pore size not only affects the osteogenic differentiation of hydrogel-bearing stem cells but also modulates the immune response of local bone tissue and promotes differentiation for the anti-inflammatory phenotype of macrophages [22].

Second, the stiffness of the hydrogels applied in craniomaxillofacial regeneration should be of concern. The stiffness of hydrogels is tunable by varying the cross-linking in the hydrogels [25,26,27], doping nanoparticles [28,29,30], and combining two different polymers to form hybrid hydrogels [15,31]; the range of the tunable stiffness of hydrogels has been reported to reach 0.5 kPa to 5 MPa [5]. As an application scenario for hydrogels, bones have a large elastic modulus of 100 kPa [32], and the stiffness of the skeletal microenvironment is an important physical factor affecting the differentiation direction of human mesenchymal stem cells (hMSCs). Stem cells have been shown to be cultured on polyacrylamide hydrogels with adjustable rigidity; in an environment with a higher Young’s modulus (i.e., a harder environment, such as a Young’s modulus of 42.1 ± 3.2 kPa [33] or 62–68 kPa [34]), various osteogenic markers of stem cells, such as runt-related transcription factor 2 (RUNX2) gene and alkaline phosphatase (ALP) staining, were upregulated in expression and osteogenic differentiation increased. Thus, methods have been developed to modulate the mechanical strength of hydrogels, such as the addition of nanoparticles.

### 2.2. Wide Variety of Drug Delivery Methods

#### 2.2.1. Physical Loading

Physical loading is the use of steric interactions of pore sizes to slow the release of drugs or to restrict the movement of cells and drug molecules. At present, there are three main theoretical models for the diffusion process of goods with different diameters in hydrogels, namely, free volume, hydrodynamic, and obstruction theory [35]. For the free volume theory, the most important thing is the ratio of the solute molecule diameter to the mesh diameter. The larger the ratio, the greater the resistance to solute molecule diffusion and the slower the diffusion rate [36,37]. According to the hydrodynamic theory, which considers the diffusion process of drug molecules and the frictional forces between the surrounding hydrogel matrix [38], the diffusion of macromolecular drugs in the hydrogel system is slowed down by the frictional forces exerted on it by the surrounding polymer chains due to the relative displacement that occurs. For cells and some macromolecular drugs, their diameters are extremely large, even larger than the mesh diameter of the hydrogel, when the obstruction theory should be considered [39]. Axpe et al. [35] integrated three theoretical models and developed the multiscale diffusion model (MSDM), which describes the diffusion process of drug molecules of different diameters from hydrogel systems with different pore sizes using mathematical models. It provides a very meaningful mathematical tool for the development of a hydrogel drug delivery system.

#### 2.2.2. Covalent Conjugation and Electrostatic Interaction

Hydrogels, with their abundant side chain groups, have become effective options for delivering small-molecule drugs, such as acetylsalicylic acid (ASA) [40] and the commonly used orthopedic drug alendronate (ALN) [41], through covalent conjugation and electrostatic interactions, which can effectively achieve bone tissue repair.

Furthermore, although covalent conjugation and electrostatic interaction are unique in delivering small molecules, both forces also show excellent properties in delivering biologically active macromolecules. Recently, Schoonraad et al., achieved the thiolation of bone morphogenetic protein 2 (BMP-2) and its covalent binding to polyethylene glycol hydrogels with the use of thiol-norbornene click chemistry, which enhanced the induced osteogenic properties of the hydrogels [42]. This type of method, by covalent binding, which can continuously exert drug effects at the tissue interface, should be considered for use in craniomaxillofacial bone regeneration. Notably, it has been suggested that, after covalent modification, bioactive molecules may be difficult to deliver to specific sites. Moreover, after extensive chemical modifications, the properties of covalently conjugated molecules may be affected [43].

Electrostatic attraction is a form of nonspecific interaction between positive and negative charges to attract, with the ability to control the transport of different drugs at the same time [44]. Electrostatic effects are often used to transport biologically active macromolecules. For example, Kolambkar et al., used alginate hydrogels with a negative charge to convey cationic, heparin-bound growth factors to facilitate tissue regeneration [45]. Electrostatic attraction delivery is also used for chemical drug delivery. A doxorubicin (DOX) delivery system was developed by Yu et al., in which positively charged antitumor drugs are bound to a negatively charged carboxyl surface by electrostatic attraction, forming NPs for stable drug delivery [46]. However, the electrostatic interactions between the components may increase the cross-link density of the hydrogel; thus, the drug release rate and hydrogel material properties should be considered.

### 2.3. Stimuli-Responsive Hydrogels That Can Be Applied to Craniomaxillofacial Bone Reconstruction

**Figure 3:**
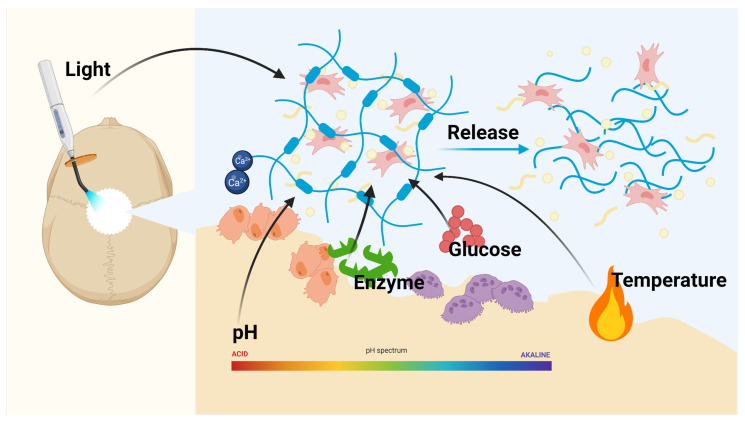
Schematic illustration of a variety of different stimulus-responsive drug delivery system. Stimulus-responsive hydrogels are subjected to specific stimuli, such as light, pH, enzymes, temperature and biochemical conditions, and decompose and deform in response to these stimuli, thereby releasing the cells and drugs. The figure was made with BioRender, accessed on 1 December 2022 (https://biorender.com/).

Delivered drugs will be more efficient if they are more adapted to the spatial, temporal, and dose requirements of the local microenvironment. The recognition of the microenvironment and response in a dynamic manner that mimics the reactivity of organisms is called the stimulus-responsive system [47]. This concept was first proposed in the late 1970s, using liposomes to achieve a temperature response to facilitate tumor thermotherapy [48]. Recently, researchers have developed a variety of smart hydrogels that can precisely respond to many stimuli, such as enzymes, temperature and light (Figure 3).

#### 2.3.1. Enzyme-Responsive Hydrogels

Drug delivery systems target the human body, in which enzymes are common bioenvironmental trigger factors. Enzyme-responsive hydrogels can be prepared by incorporating target enzyme-specific recognition fragments into the hydrogel network and further controlling drug release through hydrogel degradation or hydrogel deformation [49,50,51]. Most of the enzyme-reactive hydrogel systems reported to date are protease- and glycosidase-based, with protease-mediating peptide bond hydrolysis and glycosidases catalyzing the polysaccharide hydrolysis [51]. Matrix metalloproteinases (MMPs) are a target of protease-responsive hydrogel delivery systems. MMPs have been found to be upregulated in inflammatory and tumor tissues and are important in promoting cancer proliferation [52]. Najafi et al., developed an MMP-responsive hydrogel embedded with peptides that can be recognized by MMPs. By embedding the substrate L-amino acid sequence of matrix MMPs 2 and 9 in the poly (ethylene glycol) (PEG)-based hydrogel network, the system gained the capacity to respond to MMPs [53].

#### 2.3.2. Temperature-Responsive Hydrogels

Thermo-sensitive hydrogels are a category of hydrogels that trigger reversible sol-gel phase transitions when stimulated by a specific temperature. Thermo-responsive hydrogels generally exhibit a lower critical solution temperature (LCST), meaning that thermally responsive hydrogels can often behave as a solution in vitro and a sol-gel transition occurs when heated to normal body temperature, forming a solid hydrogel [54]. Poly(N-isopropylacrylamide) (PNIPAAm) is a temperature-responsive polymer widely used to prepare thermo-sensitive hydrogels, but it has disadvantages, such as poor biodegradability and extremely rapid release, which limit its applications in drug delivery [55]. PEG cross-linked PNIPAAm can significantly enhance the properties of hydrogels [56]. Johnson et al., prepared a thermo-sensitive hydrogel with a three-dimensional structure based on PEG/PNIPAAm, while they innovatively explored a method to optimize the PEG/PNIPAAm hydrogel system. By elucidating the pendant alkyl groups on the polymer in influencing the LCST of the hydrogel, they proposed that the LCST gradually decreases with an increasing amount of n-butylamine, which serves as a precious source for the development of appropriate PEG/PNIPAAm-based hydrogels [57].

#### 2.3.3. Light-Responsive Hydrogels

Photoreactive hydrogels are a class of hydrogel systems in which light modulates the cross-linking and drug release of hydrogels, usually consisting of polymers modified with photosensitive functional groups cross-linked under light irradiation [51,58]. In recent years, near-infrared (NIR) light has attracted attention from researchers because of its reduced photodamage and better tissue penetration [59]. Yan et al., first used upconverting NPs (UCNPs), which induced a gel–solution transition upon irradiation by NIR light at 980 nm. These NPs could convert NIR photons to ultraviolet (UV) photons, possibly leading to hydrogel degradation, initiating the release of encapsulated biomolecules [60].

In addition, many optogenetic tools have recently been applied to the design of photosensitive hydrogels [61,62]. Duan et al., developed a light-responsive hydrogel through a light-responsive cross-linking mechanism called LOVTRAP. The noncovalent binding between light-oxygen-voltage-sensing domain 2 (LOV2) and ZDark1 (zdk1) has a much higher binding affinity under blue light irradiation than in the dark; thus, the dissociation between LOV2 and zdk1 occurs when exposed to blue light conditions. Under dark conditions, LOV2 binds to zdk1 and increases cross-linking of the hydrogel [63].

#### 2.3.4. pH-Responsive Hydrogels

pH-responsive hydrogels release or receive protons depending on the pH in the microenvironment, and then release the loaded drug by deformation or electrostatic attraction [64]. Studies on some polymers that can respond to pH changes have inspired pH-responsive hydrogels, e.g., polycations tend to undergo smaller swelling in neutral environments, poly(N,N’-diethylaminoethylmethacrylate) ionizes at low pH, and poly(acrylic acid) can ionize in alkaline environments [65,66]. Yu et al., synthesized a novel anionic nanomaterial poly(methacrylate citric acid) (PCA) for producing pH-responsive hydrogel. PCA, a polymer with the ability to chelate Cu^2+^ and that exhibits pH responsiveness, is being used as a candidate for the pH-responsive hydrogel delivery of chemotherapy drugs [46].

## 3. Smart Hydrogels: Application in Craniomaxillofacial Bone Engineering

**Figure 4:**
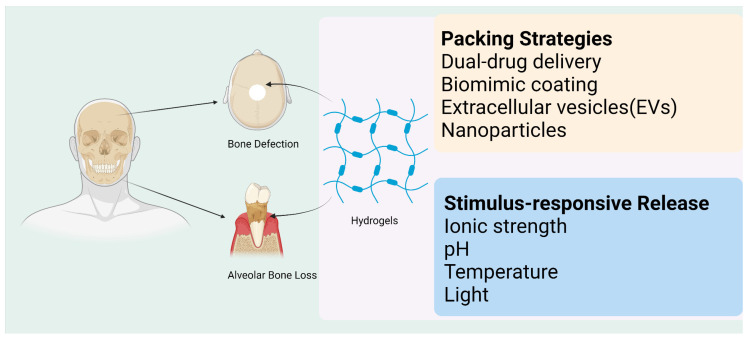
Hydrogels have been developed via different perspectives of bone repairing. Hydrogels pack different drugs and cells to serve as a scaffold in tissue engineering. The functional groups, forming part of the hydrogels, also contribute to drug loads. Responsive systems can also be applied in promoting the reconstruction of craniomaxillofacial bone defects. This image was drawn using BioRender, accessed on 1 December 2022 (https://biorender.com/).

As discussed in Section 2, the structure and the ability to load and deliver cargoes intelligently, which hydrogels possess to properly adapt to the microenvironment of bone defects, have led hydrogel systems to be considered as potential candidates for applications in the craniomaxillofacial region. Researchers have made many efforts to promote their clinical application (Figure 4).

### 3.1. Appropriate Structure for Carrying Cargo

As discussed in Section 2.1, the various material properties of hydrogel systems facilitate their use in the repair of craniomaxillofacial defects. First, the proper pore size is an important requirement for applications as a delivery system for craniomaxillofacial defects. The current hydrogel pores tend to achieve optimal encapsulation efficiency for drugs and cells by modulating the cross-linking density. In addition, appropriate pores can provide hydrogel microspheres with a larger surface area. The porous Ca-Gellan gum (GG) hydrogel microspheres designed by Zhang et al., reached a 74.14 ± 2.27% porosity, which can effectively encapsulate interleukin-4 (IL-4) [67]. It is worth mentioning that changes in the degree of cross-linking sometimes bring about changes in the macroscopic morphology. This feature, if controlled, can have interesting applications in tissue engineering. Recently, Fraser et al., creatively applied the swelling-mediated biomechanical properties of hydrogel systems to ingeniously align periodontal ligament cells (PDLCs) with the dentin surface, enabling a more physiological local pro-healing environment and representing a potential approach to overcome the challenges of achieving native dentin restorations in dentistry [68].

In addition, the proper stiffness is important to match the microenvironment of craniomaxillofacial bone defects. Hydrogels applied to craniomaxillofacial bone defects tend to modulate stiffness by incorporating nanoparticles and by introducing cross-linked networks. Due to the different interactions between different nanoparticles and polymer networks, the ability of different nanoparticles to enhance the mechanical strength varies and should be selected according to the context of clinical applications. Zhou et al., enhanced the mechanical properties of the hydrogel by adding MgCO_3_ and MgO nanoparticles to the hydrogel network and also showed that although the addition of both nanoparticles enhanced the Young’s modulus of the hydrogel network, the addition of MgCO_3_ particles (45.20 ± 7.93 MPa) increased the hardness of the hydrogel more significantly [69]. The introduction of a hybrid double-cross-linked hydrogel can likewise provide the proper stiffness for the hydrogel system. Li et al., developed a borax ion cross-linked double-network hydrogel that significantly enhanced the stiffness of the hydrogel system. In addition to ensuring matrix stiffness, they loaded the hydrogel with demineralized bone matrix (DBM) powder and hypoxia-pretreated bone marrow stromal cells. This highly restored the extracellular matrix (ECM) environment in bone and produced good repair results in a 7 mm diameter critical cranial defect in rabbits [15].

### 3.2. Smarter “Packing” of a Wide Range of Cargoes

As previously explained, hydrogel systems possess a variety of unique properties suitable for use in craniomaxillofacial bone tissue engineering; using hydrogels in drug delivery scaffolds is one of the best options for delivering cargoes in the craniomaxillofacial region. For this reason, there is interest in exploring how to match the drugs loaded more intelligently to achieve the best healing effect (Table 1). One of the major advantages of physical loading, as a nonspecific interaction with drugs, is the possibility of carrying many different species of drugs. The goal of dual and sequential drug delivery can be achieved through physical loading. Park et al., developed an alginate/collagen-based hydrogel in which microparticles (MPs) packed with insulin-like growth factor 1 (IGF-1) and BMP-2 were loaded into the hydrogel system. The different degradation rates of the gelatin MPs (gMPs) and PLGA-PEG-COOH MPs (pMPs) led to the sequential release of BMP2 and IGF1, resulting in excellent bone regeneration in rat cranial defects of up to 8 mm in diameter [70].

Nano-hydroxyapatite (nHA) is a bioceramic material that has been proven to have the ability to promote bone tissue healing [76,77]; however, nHA has the disadvantages of insufficient mechanical strength for inducing osteogenesis [78]. With its editability and biodegradability, the hydrogel can be used to achieve the intelligent delivery of nHA. Pan et al., prepared a hydrogel–hydroxyapatite (GH) scaffold and successfully induced bone regeneration in a rat alveolar bone defect model with extracted mandibular central incisors. Interestingly, the results of Pan et al.’s work explored the effect of their composite materials in preserving the height of the alveolar ridge around the alveolar socket after tooth extraction, significantly achieving strong aesthetic healing of the mandibular central incisor extraction socket in rats [72]. The aesthetic healing effect is very important during dental treatment, and the evaluation of the height of the alveolar ridge by Pan et al., is very meaningful.

The important role of metal ions in promoting osteogenesis and angiogenesis is gradually being elucidated [79]. Modification of the hydrogel surface function facilitates the intelligent carrying of metal oxides and enables the controlled release of metal ions. Zhou et al., demonstrated an injectable MgO/MgCO_3_@PLGA (PMM) hydrogel and proposed that the sustainable release of magnesium ions could be regulated by modulating the mass ratio of MgO and MgCO_3_ incorporated into the hydrogel system [69]. Chen et al., firstly phosphocreatine-functionalized chitosan and then used phosphate groups in phosphocreatine-functionalized chitosan (CSMP) through metal–ligand supramolecular binding with MgO NPs, which achieved the controlled release of magnesium ions in the local environment [73].

Hydrogels are likewise candidates for transporting extracellular vesicles (EVs), which have been shown to play a role in bone regeneration [80,81,82]. Shen et al., introduced a dental pulp stem cell (DPSC)-derived exosome-loaded hydrogel system, which promoted alveolar bone regeneration in periodontitis mice. The application of DPSC-derived exosome regulated the immune response and promoted the shift of macrophages to anti-inflammatory macrophages [74]. Liu et al., similarly suggested that macrophages in the periodontitis environment can also be modulated in polarization by EVs derived from bone marrow mesenchymal stem cells (BMMSCs). Liu et al., prepared a hydrogel with relatively precise controlled release and biocompatibility with gelatin and loaded EVs derived from BMMSCs to promote periodontal bone recovery in a rat with periodontitis [75].

Furthermore, as previously mentioned, BMP-2 can be firmly tethered to the hydrogel surface by covalent bonds, and hydrogels can be modified on their surfaces using their abundant side chain groups to achieve surface bionic camouflage to accurately mimic the healing environment of bone defects. A glycopeptide hydrogel (GRgel) formed by β-sheet RADA16-grafted glucomannan self-assembly was developed by Wang et al., for the formation of a bionic camouflage surface of polycaprolactone/nHA (PCL/nHA) scaffold for good cranial repair [16].

### 3.3. Responding More Intelligently to “Deliver” Cargoes

Although many of the response methods used in delivering chemotherapy drugs may not be suitable for application in the bone healing process due to the microenvironment of bone trauma and lack of blood supply, many smart responsive hydrogel systems have been developed by innovative researchers to achieve the effective healing of cranial bone defects with regard to the characteristics of the local environment of cranial bone defects (Table 2).

In an environment of bone defects, due to the destruction of bone, inorganic mineral components in bone tissue composition are released in ionic form, and the concentration of some ions in the local environment increases, which enables the development of ion-strength responsive hydrogels. Zhang et al., developed a Ca–GG hydrogel microsphere based on GG hydrogel, a polysaccharide-based biomaterial with good biodegradability that has been approved by the FDA for application, in which Ca^2+^ can respond to free phosphate ions at the location of the local bone defect and can precipitate locally and further subsequently mineralize [67].

Similarly, during the bone healing procedure, the local microenvironment in the regeneration site is accompanied with unique characteristics, such as changes in the pH environment and in the expression of various enzymes and proteins. Bai et al., developed a pH-responsive hydrogel compounded with bio-glass (BG), based on different cross-linking strategies, such as acylhydrazone bond cross-linking and DA click covalent cross-linking, empowering the system’s ability to degrade under alkaline conditions. As the hydrogel system degraded, BG exposure and the release of Ca^2+^ promoted local bone repair. At the same time, BG exposure and combination with surrounding tissues will increase the pH of the local environment, further promoting the degradation of the hydrogel system and the exposure of BG, finally releasing Ca^2+^. In vivo experiments applied to a 5 mm critical size bone defect in rat skull showed that the hydrogel effectively promoted bone repair with a higher repair capacity than a similar hydrogel system encapsulating BMP-4 [83]. Silica-based NPs, with good osteoconductivity and osteoinductivity properties, can endow the material doped with silica-based NPs good performance in response to local bone deposition. Based on a dual network, Zhang et al., used soft GelMA stiff nanocomposite (NC) hydrogels, covalently bonded polyhedral oligomeric silsesquioxanes (POSS) blocks to a methacrylated chitosan (CSMA) network through photocross-linking to pack cells for effective healing in critical bone defects in the rat cranium [84].

Temperature-responsive systems that produce intelligent responses to physiological body temperature conditions are always of concern in the field of biomedicine, and the application of craniomaxillofacial tissue defect regeneration is no exception. Ai et al., designed a temperature-responsive PEG-dithiothreitol (DTT) hydrogel with an innovative loading of AM1241, which strongly regulated the balance between bone formation and resorption in the bone defect, enabling a critical bone defect in the rat skull to be well repaired [85]. Diniz et al., utilized the thermally responsive novel hydrogel carrier pluronic F-127 (PEO^®^100PPO65PEO100), which exhibited unique cross-linking properties as well as micelle morphological characteristics in a body temperature (37 °C) environment. Diniz et al., used pluronic F-127 loaded with human recombinant BMP-4 (rhBMP-4), loaded with DPSCs, which greatly enhanced the photobiomodulation (PBM) therapy, achieving good bone repair in a 4.3 mm diameter cranial defect in nude mice [18].

Moreover, photoreactive hydrogels are also worthy of attention in oral and maxillofacial bone tissue engineering. Gan et al., selected the noncoding microRNA Chol-miR-26a, a cholesterol-modified microRNA, which can be used to promote osteogenic differentiation of hMSCs and can be covalently patterned onto PEG hydrogels by UV-cleavable ester bonds. Chol-miR-26a was released after exposure to UV light, significantly enhancing alkaline phosphatase activity and repairing critical bone defects in a rat cranial bone defect model [87].

## 4. Discussion

In the process of craniomaxillofacial tissue repair, the design of hydrogels should consider the craniomaxillofacial tissue environment; this study presents issues to consider in the design of materials during craniomaxillofacial bone tissue repair. (i) Considering the skeletal microenvironment, a hydrogel matrix applied to craniomaxillofacial defects should ensure sufficient stiffness to match the surrounding skeletal microenvironment. Methods to enhance stiffness include increasing the cross-linking [25,26,27], doping with nanoparticles [28,29,30], and creating hybrid hydrogels [15,31]. Increasing the degree of cross-linking is often accompanied by a decrease in mesh size [37], which can affect the ability of the hydrogel to load drugs and cells; therefore, there is a limit to the effectiveness of increasing the degree of cross-linking to improve stiffness. The use of nanoparticles and the preparation of hybrid hydrogels are often more popular because of the introduction of new components in the hydrogel system, such as the introduction of borax ions to prepare double-cross-linked network hydrogels [15] and the addition of nano-hydroxyapatite (nHA) [72]. (ii) When considering the addition of nanoparticles, the unique properties of nanoparticles should be considered, such as gMPs and pMPs with different degradation rates for sequential drug release in hydrogel delivery systems [70] and the loading of metal oxides for the continuous delivery of Mg^2+^ [69,73]. In addition, studies on functionalized nanoparticles should be discussed; for example, NIR has better tissue penetration [59], and even the potential of NIR to induce osteogenic differentiation of MSCs has been reported [88], and its ability to penetrate the patient’s scalp to irradiate cranial defects in clinical practice deserves to be evaluated. NIR likewise provides inspiration as to whether upconverting nanoparticles can be introduced for the preparation of NIR-responsive hydrogel systems. (iii) In the process of designing materials to promote bone defect healing, it should always be remembered that the repair of craniomaxillofacial bone tissue defects is a physiological process and that the repair process of craniomaxillofacial bone tissue is essentially a modulation of the various actors involved in this physiological process, such as the use of EVs [74] or the design of materials [67,89] to modulate macrophages and induce them to polarize toward a pro-repair phenotype. Bone regeneration is a complex process in which multiple cellular and signaling pathways interact with each other [90], and the modulation of this physiological process from either perspective has the potential to produce results that favor the healing of bone defects. RNA regulation, for example, can be achieved by introducing mRNA, miRNA, or siRNA to the scaffold to regulate the local cellular expression of proteins required for repair and to reduce the expression of proteins detrimental to repair [91]. The introduction of chol-miR-26a, for example, can provide the required miRNA locally in bone defects for a long time [87]. (iv) How the material is loaded with drugs is equally worthy of discussion. In this paper, we summarized several key methods of loading hydrogels with cargo, and the choice of which method to use to load drugs is an issue that must be considered in the material design process. For small-molecule drugs, physical loading methods cannot be used because small-molecule drugs will follow free volume theory in the hydrogel network and lead to rapid diffusion [36]; therefore, small-molecule drugs should mostly be loaded by means of covalent conjugation and electrostatic interactions, such as ASA [40] and ALN [41]. In addition, molecules such as modified BMP-2 [42] and chol-miR-26a [87] can be grafted on the hydrogel surface by covalent conjugation, thus sustaining their role at the bone defect repair interface. It is worth discussing which drug treatment is better for modified molecules versus unmodified molecules and tethered molecules versus free molecules. (v) Craniomaxillofacial bone defects are often irregular in shape, and the best option for good shape restoration is to use injectable hydrogel. However, injectable hydrogels are limited in that they can clog syringes if they gel too quickly, and it is difficult to gel them in a timely manner if they gel too slowly. The modification of injectable hydrogel systems using appropriate methods is something that should be considered, such as the application of borax ions to construct double-cross-linked networks [15,92], DNA-based covalent imine bonds [30], and enzymatic cross-linking [93] to prepare self-healing injectable hydrogels. Before hydrogels can be used clinically in craniomaxillofacial bone tissue repair, it is necessary to select a suitable method to yield good injectability of the hydrogel.

## 5. Conclusions and Perspective

Due to the fact of their modifiable physicochemical properties, hydrogels have shown tremendous influence in several fields, such as tissue engineering [13,14], treatment of cancer [46,94], and even biosensor development [95]. This review outlined the unique physicochemical properties of the hydrogel system itself, including the two important characteristics of stiffness and pore diameter, as well as the loading method and stimulus-response system, and thus summarized the hydrogel systems that have recently been reported for application in the bone regeneration of craniomaxillofacial bone tissue. As previously discussed, hydrogel systems should be designed considering the environment of their application. For example, in craniomaxillofacial tissue repair, consideration should be given as to whether the stiffness of the designed hydrogel is adapted to the local skeletal microenvironment, whether the pore size is well suited to the cells or drugs being loaded, whether a proper approach is taken to load the cargo, and whether the local microenvironment is appropriately and intelligently responded to.

Despite the achievements of hydrogel systems in the field of craniomaxillofacial bone tissue repair, it is still worth exploring in the future whether the intelligence of hydrogel systems can be optimized even more. For example, as mentioned previously, it is worth exploring whether the physiological effects of NIR-responsive hydrogels combined with NIR will achieve a good effect in promoting skull healing. In addition, MMPs are often elevated in the inflammatory environment accompanying bone defects, and it is worth considering whether the MMP-sensitive hydrogel developed by Najafi et al. [53], relying on the L-amino acid sequence, can be applied in response to the inflammatory environment in which the bone defect is located. In addition, it is known that the pH environment inside the oral cavity is often influenced by eating, and the pH of the oral environment is easily adjusted; therefore, it is also worth exploring the development of pH-responsive hydrogels for oral applications based on this property. Similarly, the oral cavity is generally in a closed and dark condition, but the environment inside the mouth can be exposed to specific light when the patient opens their mouth or when the operator uses a specific light source. LOVTRAP, employed by Duan et al. [63], can respond well to this environment, and optogenetic tools are well worth being considered for application in oral and maxillofacial regions. All of these suggest that, in the future, smarter hydrogel systems being applied to craniomaxillofacial bone tissue engineering need to be developed considering multidisciplinary collaboration.

## Figures and Tables

**Table 1:** Summary of the different strategies for carrying cargoes to enhance the cure therapy.

Strategies	Carriers	Cargoes	Reference
Dual drug delivery	Double-crosslinking architecture of borax-mixed alginate dialdehyde (ADA) and gelatin	Demineralized bone matrix (DBM) powder (mimicking the ECM environment of bone trauma) and hypoxia-pretreated bone marrow stromal cells	[15]
	>Aginate/collagen-based hydrogel	gMP-BMP2, pMP-IGF1	[70]
Nanoparticles	Poly (lactic-co-glycolic acid) (PLGA)-dextran (PLGA-Dex)	Nano-hydroxyapatite (nHA)	[71]
	Chitosan/hyaluronic acid-aldehyde hydrogel	Injectable soft self-repairing hydrogel–hydroxyapatite scaffold	[72]
	PLGA	MgO/MgCO_3_	[69]
	Phosphocreatine-functionalized chitosan (CSMP)	MgO	[73]
Extracellular vesicles (EVs)	Chitosan-based hydrogel	Dental pulp stem cell (DPSC)-derived exosomes	[74]
	Gelatin	Bone marrow mesenchymal stem cells (BMMSCs)	[75]
Biomimic coating	Glycopeptide hydrogel	Self-assembly of β-sheet RADA16-grafted glucomannan	[16]

**Table 2:** Summary of stimulus-responsive systems and methods to be able to respond to different stimulus.

Stimulus-Responsive System	Methods	Functional Block	Reference
Ion-strength	Ca^2+^ can respond to free phosphate ions at the local bone defect	Ca–gellan gum (GG) hydrogel	[67]
pH	Acylhydrazone bond cross-linking and DA click covalent cross-linking	Bio-glass (BG)	[83]
	Changes in osteoconductivity and osteoinductivity	Silica-based NPs	[84]
Temperature		PEG-dithiothreitol (DTT)	[85]
		Pluronic F-127 (PEO^®^100PPO65PEO100)	[18]
		Calcium lactate (CaL)	[86]
Light	UV-cleavable ester bond	Cholesterol-modified noncoding microRNA Chol-miR-26a	[87]

## Data Availability

Not applicable.
